# Cost-effectiveness of an artificial intelligence predictive model for guiding androgen deprivation therapy in intermediate-risk prostate cancer

**DOI:** 10.1093/jncics/pkag035

**Published:** 2026-04-09

**Authors:** P Travis Courtney, Ya-Chen Tina Shih, Albert J Chang, Alan Lee, Michael L Steinberg, Luca F Valle, Puja S Venkat, Amar U Kishan, Ann C Raldow

**Affiliations:** Department of Radiation Oncology, University of California, Los Angeles, CA, United States; Department of Radiation Oncology, University of California, Los Angeles, CA, United States; Department of Radiation Oncology, University of California, Los Angeles, CA, United States; Department of Radiation Oncology, University of California, Los Angeles, CA, United States; Department of Radiation Oncology, University of California, Los Angeles, CA, United States; Department of Radiation Oncology, University of California, Los Angeles, CA, United States; Department of Radiation Oncology, University of California, Los Angeles, CA, United States; Department of Radiation Oncology, University of California, Los Angeles, CA, United States; Department of Radiation Oncology, University of California, Los Angeles, CA, United States

## Abstract

The ArteraAI Prostate Test (ArteraAI Inc.) is the first predictive biomarker for the benefit of adding short-term androgen deprivation therapy (ADT) to radiotherapy for intermediate-risk prostate cancer. We evaluated the cost-effectiveness of ArteraAI to guide short-term ADT using a Markov model simulating 15-year outcomes for 71-year-old patients with intermediate-risk prostate cancer receiving radiotherapy using NRG/RTOG 9408 data on which ArteraAI was validated. Three strategies were compared: (1) all patients receive ADT (ADT-for-all), (2) only patients with unfavorable intermediate-risk prostate cancer receive ADT (National Comprehensive Cancer Network [NCCN]), and (3) only ArteraAI-positive patients receive ADT (ArteraAI). Costs and utilities obtained from Medicare claims and published literature were used to calculate incremental cost-effectiveness ratios. A willingness-to-pay threshold of $100 000/quality-adjusted life year (QALY) was chosen. The ADT-for-all strategy was dominated by the NCCN strategy. Compared with the NCCN strategy, the ArteraAI strategy lowered costs by $12 296 and improved effectiveness by 0.01 QALYs making it dominant.

In patients with intermediate-risk prostate cancer undergoing definitive treatment with radiotherapy, the addition of 4-6 months of androgen deprivation therapy (ADT) has been shown to reduce the risk of distant metastasis, prostate cancer-specific mortality, and death on a population basis.^1^ However, even with short-term ADT, many patients experience significant side effects,^2^ which may decrease health-related quality-of-life and increase health-care-related costs. Strategies to identify patients who may benefit from the addition of short-term ADT with radiotherapy have been proposed, most notably according to National Comprehensive Cancer Network (NCCN) risk groups, whereby patients with unfavorable intermediate-risk prostate cancer are recommended to receive short-term ADT, whereas those with favorable intermediate-risk prostate cancer are not,^3^ although data suggest that NCCN risk grouping is not predictive of ADT benefit.^1^ Similarly, multiple biomarkers have been developed to address this question and demonstrated prognostic ability, though none have been shown to be predictive of benefit from ADT.^4^ Because such, there is a pressing clinical need for more accurate risk stratification tools to guide short-term ADT use in this patient population.

A multimodal artificial intelligence-based biomarker (ArteraAI Prostate Test, ArteraAI Inc.) is the first AI-enabled biomarker test to predict the benefit of adding short-term ADT to radiotherapy in patients with intermediate-risk prostate cancer.^5^ The ArteraAI Test was trained on multiple historical randomized controlled trials and validated on NRG/RTOG 9408, which randomized patients with predominantly intermediate-risk prostate cancer to radiotherapy with or without 4 months of ADT.^6^ Among ArteraAI-positive patients, those who received ADT had significantly lower rates of distant metastasis and prostate cancer-specific mortality, whereas among ArteraAI-negative patients, there were no significant differences in these outcomes by ADT use.

Given the high incidence of prostate cancer, incorporating biomarker tests into the standard of care has tremendous economic implications, considering the costs associated with the test and downstream events. We evaluated the cost-effectiveness of using the ArteraAI Test to guide the use of short-term ADT for intermediate-risk prostate cancer vs using NCCN risk grouping^3^ or giving short-term ADT to all patients with unfavorable intermediate-risk disease.

We followed Consolidated Health Economic Evaluation Reporting Standards (CHEERS) guidelines for cost-effectiveness analyses.^7^ This study was exempted from University of California, Los Angeles institutional review board review because it used published trial data.

We compared 3 strategies to guide the use of short-term ADT: (1) all patients with intermediate risk receive ADT (ADT-for-all), (2) only patients with NCCN unfavorable intermediate-risk prostate cancer receive ADT (NCCN), and (3) only ArteraAI-positive patients receive ADT.

We created a Markov model in which patients with newly diagnosed, intermediate-risk prostate cancer undergoing radiotherapy with or without short-term ADT could transition through 4 health states: (1) stable/no evidence of disease on ADT (for those assigned to receive ADT), (2) stable/no evidence of disease off ADT, (3) metastatic disease, and (4) death. Patients entered the model in the stable/no evidence of disease state on or off ADT and could remain there or experience metastatic disease or death. The proportion of patients receiving ADT varied by the strategies described above (Table S1). We assumed a patient age of 71 years because that was the median age in NRG/RTOG 9408,^8^ a 1-year cycle length with a half-cycle correction and a 15-year time horizon.

Transition probabilities for metastases were obtained from the low-intermediate-risk subgroup in the ArteraAI Test validation publication^5^ and NRG/RTOG 9408 secondary analyses.^9^ We assumed equal overall survival across all strategies because compared with other strategies, the major benefit of the ArteraAI test is quality-of-life improvement from reduction of unnecessary ADT use.

In the base case model, dosages used to calculate ADT cost were derived from the NRG/RTOG 9408 regimen^6^ (4 months of oral Flutamide [250 mg] thrice daily and either monthly subcutaneous Goserelin [3.6 mg] or intramuscular Leuprolide [7.5 mg]). We tested additional ADT regimens in sensitivity analyses, namely, 4-6 months of intramuscular Leuprolide and 4-6 months of Relugolix. Of note, the cost of the NRG/RTOG 9408 regimen was largely driven by the cost of Flutamide. Radiotherapy costs were assumed to be equal in all strategies and thus not included.

We considered costs from the payer perspective. Provider-administered drugs were calculated becasue the average sales price with a 6% markup, and patient-administered drugs were obtained from the RED BOOK. Given higher end-of-life costs with cancer vs non-cancer,^10^ end-of-life costs in each strategy were calculated proportionally to each strategy’s 15-year risk of prostate cancer–specific mortality for the subset of patients who died within 15 years. Costs were adjusted to March 2025 US dollars. Effectiveness was measured as quality-adjusted life years (QALYs). Health utilities were obtained from published literature. Patients were assigned different health utilities based on the receipt of ADT.^11^ The effect of ADT on health utility was assumed to last 1 year after initiation, following known trends in testosterone recovery.^12^ A 3% annual discount rate was applied to all costs and QALYs. Table S1 shows all model inputs.^5^^,^^9-11^^,^^13-17^

We calculated the incremental cost-effectiveness ratios (ICERs, in $/QALY) and net monetary benefits (NMB) in 2025 US dollars, whereby strategies with higher NMBs are cost-effective compared with strategies with lower NMBs (ie, incremental NMBs > 0).^18^ The 3 strategies were compared using stepwise comparison. We chose a willingness-to-pay (WTP) threshold of $100 000/QALY. We performed 1-way and probabilistic sensitivity analyses using a Monte Carlo simulation with 100 000 samples. The model was created and analyzed with TreeAge Pro Healthcare 2024.

Base model results are provided in Table 1. The ADT-for-all, NCCN, and ArteraAI strategies had costs of $56 637, $46 892, and $34 596 and QALYs of 6.90, 6.92, and 6.93, respectively. In stepwise comparison, ADT-for-all was dominated by the NCCN strategy and thus not included in the final comparison. Compared with the NCCN strategy, the ArteraAI strategy lowered costs by $12 296 and improved effectiveness by 0.01 QALYs, thus dominating the NCCN strategy. Similarly, the incremental NMB for the ArteraAI vs NCCN strategy was $13 324.

**Table 1.** Incremental cost-effectiveness ratios and net monetary benefits for the 3 short-term ADT strategies.

Strategy	Cost ($)	Incremental Cost ($)	Effectiveness (QALY)	Incremental effectiveness (QALY)	ICER ($/QALY)	Net monetary benefit ($)	Incremental NMB ($)
ADT-for-All^a^	56 637	—	6.90	—	—	636 203	—
ADT by NCCN Risk Grouping	46 892	—	6.92	—	—	646 670	—
ADT for ArteraAI-Positive	34 596	−12 296^b^	6.93	0.01^b^	−1 247 000	659 256	13 324^b^

Abbreviations: ADT = androgen deprivation therapy; ICER = incremental cost-effectiveness ratios; NCCN = National Comprehensive Cancer Network; NMB = net monetary benefits; QALY = quality-adjusted life year.

a Dominated by ADT by NCCN risk grouping strategy in stepwise comparison.

b Between the NCCN and ArteraAI strategies.

Figure 1 shows the tornado diagram of 1-way sensitivity analyses comparing the NCCN and ArteraAI strategies. The model was modestly sensitive to the probability of being ArteraAI-negative in the ArteraAI strategy or having favorable intermediate-risk disease in the NCCN model, as well as the cost of ADT, which was tested across the NRG/RTOG 9408 regimen and 4-6 months of intramuscular Leuprolide or Relugolix; however, there were no scenarios in which the ArteraAI strategy became not cost-effective compared with the NCCN strategy on 1-way sensitivity analyses. The model was insensitive to all other inputs, including the ArteraAI Test price under Medicare or commercial insurance prices. Probabilistic sensitivity analysis found that at a WTP threshold of $100 000/QALY, the probability of the ArteraAI strategy being cost-effective compared with the NCCN strategy was 55.1%. At WTP thresholds of $50 000/QALY and $150 000/QALY, the probabilities that the ArteraAI strategy is the most cost-effective were 60.3% and 53.3%.

**Figure 1.**
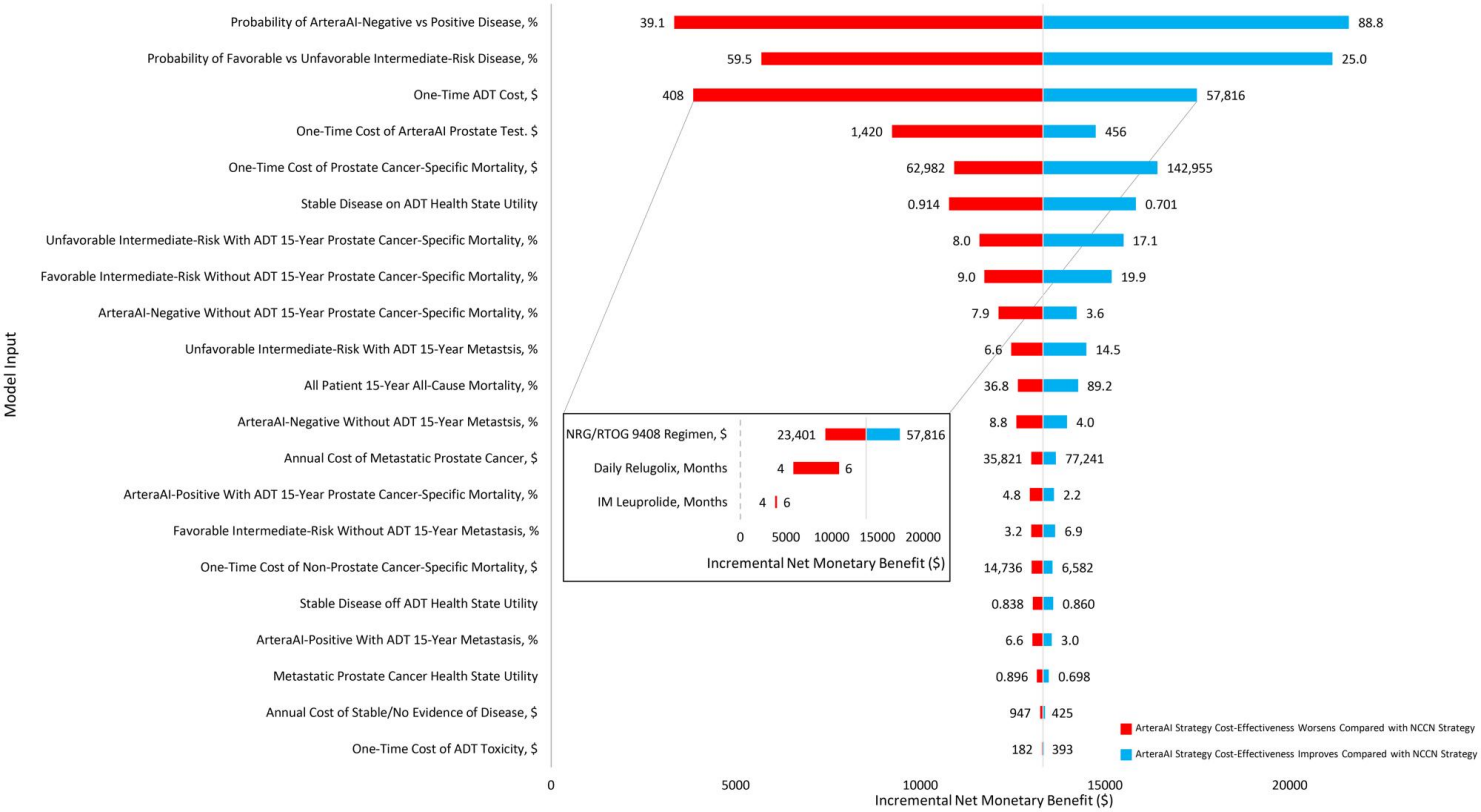
Tornado diagram of 1-way sensitivity analyses comparing the ArteraAI strategy vs the National Comprehensive Cancer Network (NCCN) strategy. The vertical gray line represents an incremental net monetary benefit of $0, above which the ArteraAI strategy would be cost-effective compared with the NCCN strategy at a willingness-to-pay threshold pf $100 000/QALY. The magnified box displays the results of the 1-way sensitivity analysis of ADT cost varied across 3 ADT regimens: NRG/RTOG 9408 (base case), 4-6 months intramuscular Leuprolide, and 4-6 months Relugolix. The cost of the NRG/RTOG 9408 regimen was largely driven by the cost of Flutamide.

In the first cost-effectiveness analysis of the ArteraAI Test, we found that using this test to guide short-term ADT use in patients with newly diagnosed, intermediate-risk prostate cancer receiving radiotherapy was the dominant strategy compared with strategies of giving short-term ADT to all patients with intermediate-risk prostate cancer or only those with unfavorable intermediate-risk disease per NCCN guidelines. Our model was robust to all inputs, supporting our conclusion that the ArteraAI Test is cost-effective in this clinical context. Probabilistic sensitivity analysis showed modest robustness, likely reflective of the small differences in effectiveness between strategies because of the numerically similar health state utilities used in our model, which were chosen from a rigorous study of a large and modern cohort of long-term prostate cancer survivors.^11^ Although the ArteraAI Test may improve quality-of-life for patients through optimization of ADT use, from the payer perspective, reduction in costs associated with ADT administration and management may drive its cost-effectiveness.

As the first and currently only predictive biomarker to guide short-term ADT use in intermediate-risk prostate cancer, the ArteraAI Test may substantially affect the current treatment paradigms in this patient population; indeed, the NCCN has listed the ArteraAI Test as an advanced risk stratification tool in their recent guidelines, although they recommend further validation before using it to guide treatment decisions in isolation.^3^ The current study argues against disuse of the test out of concern for low cost-effectiveness, given the potential for spending reductions because of fewer patients being recommended short-term ADT, provided the percentage of patients who are ArteraAI-positive remains lower than the percentage of patients who have unfavorable intermediate-risk disease outside of the NRG/RTOG 9408 validation cohort. Additional prospective validation of the ArteraAI Test in contemporary cohorts is eagerly awaited.

This study has multiple limitations. Transition probability data were derived from 2 sources evaluating different NRG/RTOG 9408 subpopulations: low-intermediate-risk in the ArteraAI Test validation publication^5^ and favorable or unfavorable intermediate-risk in the NRG/RTOG 9408 secondary analysis.^9^ The model did not include biochemical or local failure because these data were not available in the ArteraAI publication, and both have been shown to be suboptimal surrogate endpoints for overall survival,^19^ though nonetheless could have cost and quality-of-life relevance in this context. Because the ArteraAI Test is the only predictive biomarker in this clinical context, we did not evaluate other biomarkers or strategies. Regarding the assumption of equal overall survival across strategies, it is possible that ArteraAI Test inaccuracies could worsen overall survival for patients with intermediate-risk prostate cancer at the population level. Based on available data, it is difficult to estimate how ArteraAI Test performance might affect this assumption. Given the rigorous methodology used for ArteraAI Test development and validation and its strong associations with prostate cancer metastasis and mortality, we do not believe that limitations in ArteraAI Test performance would meaningfully affect overall survival for patients with intermediate-risk prostate cancer at the population level, and subsequently, our assumptions surrounding overall survival in the current study. The ArteraAI Test is expected to enhance identification of appropriate short-term ADT omission candidates whereas maintaining the overall survival rates observed with other ADT omission strategies. Finally, whereas we considered costs from the payer perspective, many came from Medicare rates; however, cost savings might be expected to be higher under commercial rates.

In conclusion, this economic evaluation found that the ArteraAI Prostate Test is cost-effective compared with NCCN risk grouping for guiding short-term ADT use in patients with intermediate-risk prostate cancer.

## Supplementary Material

pkag035_Supplementary_Data

## Data Availability

Data including cost-effectiveness models from TreeAge software will be shared upon request.
