# Supplementary material for: Cost-effectiveness of an artificial intelligence predictive model for guiding androgen deprivation therapy in intermediate-risk prostate cancer
Source: JNCI Cancer Spectr. 2026 Apr 9;10(2):pkag035. doi: 10.1093/jncics/pkag035 (PMC13127416; doi:10.1093/jncics/pkag035)
Supplement: pkag035_Supplementary_Data [file pkag035_supplementary_data.zip › ArteraAI CEA 2nd Revisions Supplementary Material Clean.docx]

**Cost-Effectiveness of an Artificial Intelligence Predictive Model for Guiding Androgen Deprivation Therapy in Intermediate-Risk Prostate Cancer**

Supplementary Material

**Table S1**: Base model inputs.

| **Parameter** | **Value** .997rm in the KEYNOTE-826 trial antsent costs **(95% Confidence Interval)^g^** | **Distribution (α**^h^**, λ/β**^i^**)** | **Reference** |
| --- | --- | --- | --- |
| ***Probabilities*** | | | |
| **% 15-Year Metastasis** | | | |
| All patients, ADT^a^ | 6.0 (3.9-8.5) | Beta  (23.4, 366.0) | Spratt et al, 2023^5^ |
| Favorable Intermediate-Risk, no ADT | 5.0 (3.2-6.9) | Beta  (23.7, 450.3) | Zumsteg et al, 2020^9^ |
| Unfavorable Intermediate-Risk, ADT | 10.0 (6.6-14.5) | Beta  (22.4, 201.6) | Zumsteg et al, 2020^9^ |
| ArteraAI Negative, no ADT^a^ | 6.3 (4.0-8.8) | Beta  (23.4, 347.3) | Spratt et al, 2023^5^ |
| ArteraAI Positive, ADT^a^ | 4.6 (3.0-6.6) | Beta  (23.8, 488.8) | Spratt et al, 2023^5^ |
| **% 15-Year Prostate Cancer-Specific Mortality** | | | |
| All patients, ADT^a^ | 4.3 (2.7-6.2) | Beta  (23.9, 531.8) | Spratt et al, 2023^5^ |
| Favorable Intermediate-Risk, no ADT | 14.0 (9.0-19.9) | Beta  (21.4, 131.2) | Zumsteg et al, 2020^9^ |
| Unfavorable Intermediate-Risk, ADT | 12.0 (8.0-17.1) | Beta  (21.9, 160.4) | Zumsteg et al, 2020^9^ |
| ArteraAI Negative, no ADT^a^ | 5.5 (3.6-7.9) | Beta  (23.6, 408.1) | Spratt et al, 2023^5^ |
| ArteraAI Positive, ADT^a^ | 3.4 (2.2-4.8) | Beta  (24.1, 690.5) | Spratt et al, 2023^5^ |
| % 15-Year All-Cause Mortality,  All patients | 65.0 (36.8-89.2) | Beta  (8.1, 4.3) | Zumsteg et al, 2020^9^, Spratt et al, 2023^5^ |
| % Favorable Intermediate-Risk^b^ | 42.5 (25.0-59.5) | Beta  (13.9, 18.9) | Zumsteg et al, 2020^9^ |
| % Unfavorable Intermediate-Risk^b^ | 57.5 (40.5-75.0) | Beta  (10.0, 7.4) | Zumsteg et al, 2020^9^ |
| % ArteraAI Negative^a^ | 66.3 (39.1-88.8) | Beta  (7.8, 4.0) | Spratt et al, 2023^5^ |
| % ArteraAI Positive^a^ | 33.7 (11.2-60.9) | Beta  (16.2, 31.9) | Spratt et al, 2023^5^ |
| ***Costs (March 2025 US Dollars)*** | | | |
| ArteraAI Test^c^ | 706 (456-1,420) | Gamma  (25, 0.04) | Personal Communication with Company |
| NRG/RTOG 9408 Regimen^d^ | 40,265  (26,475-57,816) | Gamma  (25, 0.001) | Average Sales or Wholesale Price^16,17^ |
| 1 Month Intramuscular Leuropolide | 155 (102-222) | Gamma  (25, 0.2) | Average Sales Price^17^ |
| 1 Month Daily Relugolix^e^ | 3,398 (2,183-4,961) | Gamma  (25, 0.01) | Average Wholesale Price^16^ |
| ADT Toxicity | 276 (182-393) | Gamma  (25, 0.09) | Bayoumi et al, 2000^13^ |
| Stable/No Evidence of Disease | 667 (425-947) | Gamma  (25, 0.04) | Chang et al, 2019^14^ |
| Metastatic Disease^f^ | 55,498  (35,821-77,241) | Gamma  (25, 0.001) | Horny et al, 2023^15^ |
| Prostate Cancer Death | 97,828  (62,982-142,955) | Gamma  (25, 0.0003) | Mariotto et al, 2020^10^ |
| Non-Prostate Cancer Death | 10,291  (6,582-14,736) | Gamma  (25, 0.002) | Mariotto et al, 2020^10^ |
| ***Health Utilities*** | | | |
| Stable/No Evidence of Disease, on ADT | 0.807 (0.701-0.914) | Beta  (1.8, 0.4) | Jeong et al, 2019^11^ |
| Stable/No Evidence of Disease, off ADT | 0.849 (0.838-0.860) | Beta  (2.4, 0.4) | Jeong et al, 2019^11^ |
| Metastatic Disease | 0.797 (0.698-0.896) | Beta  (1.7, 0.4) | Jeong et al, 2019^11^ |
| Death | 0 | NA | NA |

^a^Low-intermediate-risk prostate cancer subpopulation from ArteraAI publication.

^b^From NRG/RTOG 9408 secondary analysis.

^c^Centers for Medicare and Medicaid Services (CMS) price; retail price for private payers is $1,420.00. One-time cost.

^d^4 months oral Flutamide 250mg thrice daily and either monthly subcutaneous Goserelin (3.6mg) or intramuscular Leuprolide (7.5mg).

^e^Includes 360mg loading dose on day 1.

^f^For patients with CMS supplemental insurance plans; annual for patients with commercial insurance plans is $71,082.96.

^g^95% confidence intervals were obtained from published literature. In cases when standard deviations were unknown, they were assumed to be 20% of the mean. Varying the unknown standard deviation from 10% to 40% of the mean did not meaningfully impact our findings.
^h^For gamma distributions, α=25 because a standard deviation of 20% of the mean was used and the formula for α=((mean value)^2^)/((0.2*mean value)^2^).

^i^λ for gamma distributions, β for beta distributions.
